# A new *Borrelia* on the block: *Borrelia miyamotoi* – a human health risk?

**DOI:** 10.2807/1560-7917.ES.2019.24.18.1800170

**Published:** 2019-05-02

**Authors:** Sally Cutler, Muriel Vayssier-Taussat, Agustín Estrada-Peña, Aleksandar Potkonjak, Andrei Daniel Mihalca, Hervé Zeller

**Affiliations:** 1 School of Health, Sport & Bioscience, University of East London, London, United Kingdom; 2 INRA, UMR BIPAR INRA, ENVA, Anses, Maisons-Alfort, France; 3 Department of Animal Health, Faculty of Veterinary Medicine, University of Zaragoza, Spain; 4 Department of Veterinary Medicine, Faculty of Agriculture, University of Novi Sad, Serbia; 5 Department of Parasitology and Parasitic Diseases, University of Agricultural Sciences and Veterinary Medicine Cluj-Napoca, Romania; 6 European Centre for Disease Prevention and Control, Solna, Sweden

**Keywords:** *Borrelia*, tick-borne infections, relapsing fever, co-infections, vector-host transmission, epidemiology, ecology, diagnosis, treatment

## Abstract

**Background:**

*Borrelia miyamotoi* clusters phylogenetically among relapsing fever borreliae, but is transmitted by hard ticks. Recent recognition as a human pathogen has intensified research into its ecology and pathogenic potential.

**Aims:**

We aimed to provide a timely critical integrative evaluation of our knowledge on *B. miyamotoi*, to assess its public health relevance and guide future research.

**Methods:**

This narrative review used peer-reviewed literature in English from January 1994 to December 2018.

**Results:**

*Borrelia miyamotoi* occurs in the world’s northern hemisphere where it co-circulates with *B. burgdorferi* sensu lato, which causes Lyme disease. The two borreliae have overlapping vertebrate and tick hosts. While ticks serve as vectors for both species, they are also reservoirs for *B. miyamotoi*. Three *B. miyamotoi* genotypes are described, but further diversity is being recognised. The lack of sufficient cultivable isolates and vertebrate models compromise investigation of human infection and its consequences. Our understanding mainly originates from limited case series. In these, human infections mostly present as influenza-like illness, with relapsing fever in sporadic cases and neurological disease reported in immunocompromised patients. Unspecific clinical presentation, also occasionally resulting from Lyme- or other co-infections, complicates diagnosis, likely contributing to under-reporting. Diagnostics mainly employ PCR and serology. *Borrelia miyamotoi *infections are treated with antimicrobials according to regimes used for Lyme disease.

**Conclusions:**

With co-infection of tick-borne pathogens being commonplace, diagnostic improvements remain important. Developing in vivo models might allow more insight into human pathogenesis. Continued ecological and human case studies are key to better epidemiological understanding, guiding intervention strategies.

## Introduction

*Borrelia miyamotoi*, which belongs to the relapsing fever clade within the *Borrelia* genus, was first described in 1994 with its detection in *Ixodes persulcatus* ticks in Japan [1]. It was named after Professor Kenji Miyamoto who initially reported this spirochaete from Hokkaido, Japan. Its potential to cause human disease was not realised until 2011 when Platonov and colleagues described a series of cases of *B. miyamotoi *infection in Russia [2].

It is now established that this spirochaete has a global distribution and co-circulates with the related agent of Lyme borreliosis (Lyme disease), *B. burgdorferi* sensu lato (s.l.), which uses the same tick species as vectors, albeit at a lower frequency [3-9]. Similar to the Lyme borreliae, where different tick species endemic to specific regions of the globe serve as vectors, *B. miyamotoi* is found in multiple tick species that reside constrained by compatible geo-ecological habitats [2-4,10-13]. 

The objectives of this review were threefold: (i) to collate the rapid expansion of research findings on *B. miyamotoi* and its ecological interactions; (ii) to review the public health significance of *B. miyamotoi* and to (iii) highlight knowledge gaps in our understanding of this microbe and its importance as a human pathogen, thus focussing direction for future research.

## Methods

**Figure 1:**
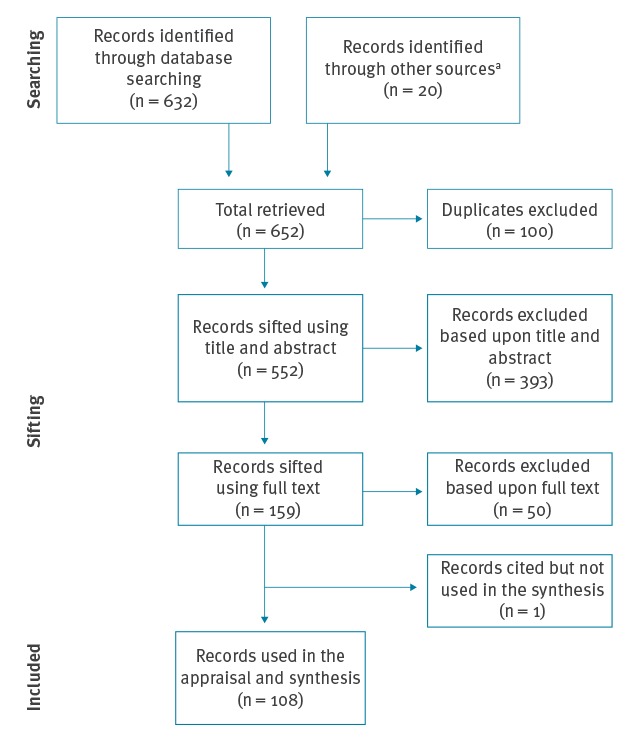
Flow chart of the search strategy and inclusion of identified articles, narrative literature review of *Borrelia miyamotoi*

We performed a non-systematic narrative literature review. Literature searches were thus not fully exhaustive. Reports relating to the search term ‘*Borrelia miyamotoi*’ published in English and indexed in biomedical databases including EBSCO (Academic Search Complete), Scopus and Science Direct were sought (Figure 1). These dated from the first description of *B. miyamotoi* in January 1994 to December 2018. Duplicate records were removed and articles were further screened, first by reading the titles and abstracts, then the full reports. Prioritisation to those papers that contributed original knowledge to our understanding was given. Supplementary literature was used to further support discussions beyond primary searches where justifiable (Figure 1).

Literature used was derived from sifting EBSCO, Science Direct and Scopus databases searching for ‘*Borrelia miyamotoi*’ from January 1994 through to December 2018.

^a ^Additional supplementary information was included as indicated to support discussion points as required. This included information on the pathobiology of other relapsing fever species.

## Results

### Epidemiology

*Borrelia miyamotoi* is being increasingly documented from the world’s northern hemisphere. This spirochaete has been recorded in Canada as well on the east and west coasts of the United States (US). It has also been observed in numerous European countries (including the Czech Republic, Denmark, Estonia, France, Germany, Netherlands, Norway, Poland, Romania, Sweden and Switzerland) as well as in Russia through to Japan [3,10,14,15]. 

Surveys to detect *B. miyamotoi* have tended to reflect local research interest rather than being systematic epidemiological studies. These have however established that endemic areas for *B. miyamotoi* overlap with those for Lyme borreliae (*B. burgdorferi* s.l.), and during tick surveys, specimens co-infected with both spirochaetes have been identified [4,11,16]. In several countries, prevalence studies based upon individual ticks infected with borreliae**have noted lower rates of ticks infected with *B. miyamotoi *than with Lyme-associated borreliae [5,17].

Among 20 studies found in this review, from countries reporting clinical cases and *B. miyamotoi *prevalence in ticks, infection**rates in ticks ranged from 0.02 to 6.4%, although most studies reported a range between 1 and 2% (Table 1) [3,5,15,18,19]. Nevertheless, pockets of higher infection rates have been described. In Napa County, California, US, for example, 15.4% of adult ticks (10/65 *I. pacificus*) studied were infected with* B. miyamotoi *[3] compared with a background infection level of 1.4% (44/3,255) of nymphs of this same species [19]. Moreover, in a study in Kurgan, Russia, 16% of ticks (26/162 *I. persulcatus*) were found infected [2]. These rates might reflect either hyperendemic areas or local efficient transmission events. 

**Table 1:** Studies worldwide reporting *Borrelia miyamotoi* infections, with diagnostic methods, prevalence and human clinical signs, 1994–2018

Location	*Borrelia miyamotoi* cases with reference (year of publication)	Percentage of cases among persons studied	Reported infection prevalence in ticks	Clinical signs	Diagnostic method	References for prevalence in ticks and human cases
**Russia**	51/302 humans bitten by ticks (2011) [2]	16.9%	*Ixodes ricinus* 0.8% *I. persulcatus* 2.9−10.5% *I. pavlovski* 6.4%	Fever, chills, sweating, headache, fatigue and vomiting (relapsing fever in 5)	PCR and serology	[2,21,104,105]
**Izhevsk, Russia**	2/24 *B. miyamotoi* infected-tick bites developed disease (2015) [58]	8.3%		Fever, chills, sweating, headache, fatigue, nausea, vomiting, dizziness.	PCR and serology	[58]
**Yekaterinburg, Russia **	71/459 tick-borne infection (including 1^a^ also with Lyme borreliosis) (2018) [22]	15.5%		Clinical details not described.	PCR and serology	[2,22]
**China**	14/984 patients with tick-borne infection (2018) [13]	1.4%	*I. persulcatus* 3% *Hyaloma concinna* 2.8%	Fever, headache, anorexia, asthenia, arthralgia	PCR	[13]
**Hokkaido, Japan **	2^a^/408 Lyme borreliosis cases (2014) [75]	0.49%	*I. persulcatus* 1.6−2% *I. pavlovski* 4.3% *I. ovatus* 0.5%	Fever, myalgia, anorexia	PCR and serology	[4,10,75]
**Japan**	12/459 suspected Lyme borreliosis (2018) [64]	2.6%		One case meningoencephalitis; clinical history not disclosed on remainder.	Serology	[4,10,64]
**Hokkaido, Japan **	1 case study (2017) [80]	NA		Fever, macular erythematous rash, low blood pressure, thrombocytopenia.	Serology	[4,10,80]
**Germany**	1 case study suspected Lyme neuroborreliosis (2016) [72]	NA	*I. ricinus* 1.2–2.4%	Lymphomatous meningitis (immunocompromised)	PCR, CXCL13 and microscopy	[3,5,72,82]
**Netherlands**	1 case study (2013) [73]	NA	*I. ricinus* 2.1–3.6%	Meningoencephalitis (immunocompromised)	Microscopy, PCR and equivocal serology	[15,73,106,107]
**Netherlands**	1 case study (2018) [103]	NA		Lymphadenopathy, leucopenia and thrombocytopenia (immunocompetent)	Serology	[15,103]
**United States **	97/11,515 acute febrile patients (2015) [78]	0.84%	*I. scapularis* 0.02−3.1% *I. pacificus* 0.4−2%	Fever, chills, myalgia, arthralgia, headaches, neutropenia, thrombocytopenia	PCR	[3,6,18,19,35,78,83,108]

NA: not applicable.

^a ^Denotes dual-infected cases who also presented with erythema migrans lesions.

In some countries, such as Mongolia, where relatively high levels of tick infections with *B. miyamotoi* (4.5%; 48/1,069 *I. persulcatus*) have been detected, no human cases were reported [10]. This was similar in the town of Hannover, Germany, where a tick-infection prevalence of 8.9% (45/505 *I. ricinus* ticks) was reported but no infections in people [20]. On the other hand, on Hokkaido Island, Japan, where, depending on the tick species, 2% (71/3,532 *I. persulcatus*) and 4.3% (5/117 *I. pavlovskyi*) of ticks were infected, human cases did occur [4]. Human cases were also observed in the Irkutsk region, Russia, where an overall 2.9% prevalence of tick infection has been estimated [21] (Table 1). In Yekaterinburg and Izhervsk, where clinical cases were first described, tick (*I. persulcatus*) infection prevalence rates of 0.9% (4/442) and 6.3% (25/394) were respectively found. Estimated incidence of human infection with *B. miyamotoi* in Yekaterinburg is likely to be 1 per 100,000 per year, accounting for a quarter of tick-borne borreliosis cases within this province [2,22].

### Phylogeny and diversity of *Borrelia miyamotoi*

**Figure 2:**
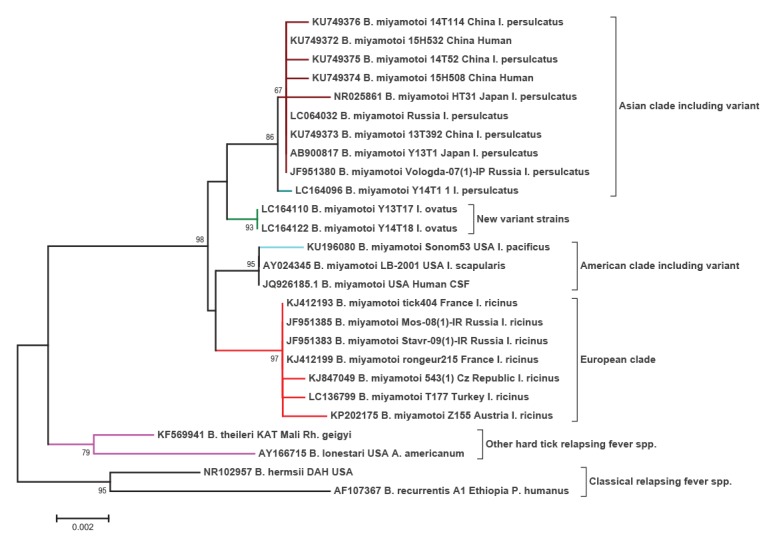
Strain diversity of *Borrelia miyamotoi* based upon 16S rRNA sequences

The taxonomic definition of the genus *Borrelia* is currently debated with the suggestion that the Lyme disease associated members be removed from this genus, and instead be placed in a new genus, *Borreliella* [23]. This suggestion has not been met with universal approval, with some experts suggesting this division is premature given the current discovery of new spirochaetes [24,25]. *Borrelia miyamotoi* clusters among the relapsing fever spirochaetes, but unlike these, resides alongside *B. theileri* and *B. lonestari* that are also transmitted by hard ticks (Figure 2).

European, American and Asian clusters are demonstrated alongside newly described strains, using classical relapsing fever species and other hard tick borreliae as outgroups. The colours of the branches denote the different clades and highlight variant strains. Tree labels provide GenBank accession numbers; strain reference; country of origin and source of sequence. The tree was compiled using a neighbour-joining algorithm, with clusters assessed using the bootstrap test for stringency (1,000 replicates). The percentages of replicate trees in which the associated isolates clustered together are shown next to the branches. The tree is drawn to scale, with branch lengths in the same units as those of the evolutionary distances used to infer the phylogenetic tree. These units represent the number of base substitutions per site. Evolutionary analyses were conducted in MEGA7.

Until recently, it was believed that *B. miyamotoi* fell into three geographically separated clades (genotypes), namely the Asian (or Siberian), European and American, (Figure 2, Table 2), transmitted by *I. persulcatus/I. pavlovskyi (I. ovatus), I. ricinus* and *I. scapularis* or *I. pacificus,* respectively [26,27].

**Table 2:** Global distribution of *Borrelia miyamotoi* clades and known vectors as at 2018

Tick species	Geographical range	Predominating *Borrelia miyamotoi* genotype (co-occurring variants)	Tick feeding preference	References
*Ixodes persulcatus*	Baltic to Far East	Asian	Generalist	[2,11]
*Ixodes pavlovskyi*	Western Siberia and Far East	Asian	Ground foraging birds, small mammals	[4,104]
*Ixodes ovatus*	South East Asia	Asian (new Asian variant)	Generalist	[4,10]
*Ixodes ricinus*	Northern Sweden to north Africa, Ireland to Ural in Russia	European (Asian)	Generalist	[11,15]
*Ixodes scapularis*	North-eastern and upper Midwestern United States	American	Generalist	[33,44,54]
*Ixodes pacificus*	Pacific coast of United States	American (new American variant)	Generalist	[44]
*Ixodes dentatus*	Eastern United States	American	Rabbits, hares Birds (larvae and nymphs)	[31]

However, as knowledge of this particular spirochaete increases, more diversity within the species is being recognised. A slight sequence variation was observed among the Asian clade based upon 16S sequence data, typified by strain Y14T1 from *I. persulcatus* ticks (Figure 2) that revealed sequence divergence from other members within this clade [10]. Similarly, in the US, divergence was noted among sequence types of American *B. miyamotoi* associated with *I. pacificus* (Figure 2) [12,28]. Given the highly conserved nature of 16S, it is not generally considered a reliable marker for the delineation of borreliae [29], as such, the level of heterogeneity among these variants might be considerably more than that inferred from 16S data alone. 

A potential fourth clade was recently described for strains found in *I. ovatus* ticks in Japan. These showed clear separation from the established sequence types for 16S rRNA (Figure 2) and the clade was further confirmed using more discriminatory multilocus sequence typing (MLST) phylogenetic approaches [30]. Representatives of this potentially novel clade of *B. miyamotoi* were carried by up to 0.6% of ticks collected from Honshu Island, but were absent from those ticks collected in Hokkaido, Japan [4,10].

Strains belonging to different genotypes of *B. miyamotoi* have been observed to geographically overlap. Asian and European genotypes have been detected in Russia and Estonia [2,11]. In south-eastern Estonia, the Asian strains were detected in both *I. persulcatus* and *I ricinus* while the European strains could only be found in *I. ricinus* [11]. 

The situation in Estonia also illustrates that a single tick species (e.g. *I. ricinus*) can harbour several genotypes [11], a phenomenon that has also been observed for *I. ovatus*, which can be infected by both the Asian and the new variant genotype [10]. On the other hand, some genotypes have been found in multiple tick species such as the American strain found in *I. scapularis* and *I. dentatus* [31], with its close variant present in *I. pacificus* [12]. Furthermore, there are reports of Asian clade representatives of *B. miyamotoi* in other types of ticks such as *Haemaphysalis concinna* [13] and *H. longicornis* [32], but the ecological role of these species for maintaining this spirochaete has not been fully elucidated. Thus vector specificity may not be as strict as previous data suggested.

### Ecology

In studies assessing single strains of *B. miyamotoi* and their associated tick species, the spirochaete has been detected among unfed larval ticks, suggesting successful transovarial (i.e. vertical) transmission to successive generations of ticks. Thus, ticks should be considered as both vectors and reservoirs for *B. miyamotoi*. Vertical transmission is estimated to have a frequency of less than 0.8 per tick generation, thus not sufficient to sustain *B. miyamotoi* for more than a few successive generations, in isolation of other transmission mechanisms [6,33]. That such mechanisms are needed has gained further support from laboratory-based *in vivo* infection studies suggesting a decline of tick infection by *B. miyamotoi* during their development, through trans-stadial moults [34]. Prevalence studies conducted with different *B. miyamotoi* tick instars revealed lower infection rates among larvae than among nymphal and adult ticks (which had equivalent rates) [35]. The higher infection rates in nymphs and adults possibly result from infection of ticks by feeding (i.e. horizontal transmission) later in their life cycle. This contrasts with a staged infection rate by instar reported for *B. burgdorferi* s.l. [11,17], whereby infection may occur predominantly by horizontal transmission (i.e. feeding).

Similar to other borreliae, prevalence of *B. miyamotoi* appears to be amplified through infection of certain avian and rodent species. Varied small vertebrate species have been shown to be competent reservoirs of infection by *B. miyamotoi* including mice (*Apodemus* spp. and *Peromyscus* spp.), voles (*Microtus* spp., *Myodes glareolus*, and *Microtus arvalis*), chipmunks (*Tamias* sp.), squirrels (*Sciuridae* spp.), European hedgehogs (*Erinaceus europaeus*) and racoons (*Procyon* spp.) [6-8,15,31,36-39]. Avian species have not been extensively surveyed, but *B. miyamotoi* has been detected in blackbirds (*Turdus merula*), great tits (*Parus major*), common chiffchaff (*Phylloscopus collybita*), song thrush (*Turdus philomelos*), European robin (*Erithacus rubecula*) and European greenfinch (*Chloris chloris*) albeit at low levels 0.6–8% of avian-removed ticks [15,31,40]. A report from Tennessee, US describes a surprisingly high level of *B. miyamotoi* infection among wild turkeys (*Meleagris gallopavo*) with 58% (35/60) birds sampled testing positive for *B. miyamotoi* [41]. These birds were heavily infested with *A. americanum* ticks (70%; 42/60), but none of the ticks were positive for *B. miyamotoi* [41]. 

Furthermore, larger vertebrates have been found with evidence of infection such as wild boar [42,43] but their ecological significance remains unclear. Interestingly, deer have been suggested to amplify *B. miyamotoi* tick infection rates in the US [44] and representatives of the Asian genotype of *B. miyamotoi* have been recovered from infected deer in the far East [32]. These observations contrast with the Lyme borreliae, where deer are not competent species to enhance transmission to ticks during feeding. Nevertheless, it must be noted that a study from Japan failed to demonstrate *B. miyamotoi* in deer, but instead reported another hard-tick vectored borrelial species resembling *B. theileri* and *B. lonestari* [45] that had been previously reported from Japanese *Haemaphysalis* spp. ticks [46] and *H. longicornis* from China [32]. A role for deer was also not corroborated by a study from the Netherlands and the European genotype [15]. Because the various studies reported here were based upon different *B. miyamotoi* genotypes, strain differences might explain the differences observed for transmission efficiency following ticks feeding upon deer. 

### Relations between* Borrelia miyamotoi* and *Borrelia burgdorferi* s.l.

In vertebrates, co-infection with *B. miyamotoi* and *B. burgdorferi* has been recorded, but the probability of dual infection appears no greater than that which would be expected independently by chance [6]. 

In ticks however, it remains a question whether co-infection happens at random or not. Some studies suggest that co-infection is more frequent than either infection alone [15]. Others however suggest that this happens by chance. For example, subsequent to a study finding 264/5,431 (4.9%) *B. burgdorferi* sensu stricto in nymphal* I. pacificus* ticks, a subset of 3,255 ticks was retested for *B. miyamotoi*. A total of 44 (1.4%) ticks were infected *B. miyamotoi* alone, and only one single individual with *B. burgdorferi* sensu stricto was also found co-infected with *B. miyamotoi* [19,47]. On the other hand, among *I. scapularis* ticks from Midwestern US, Hamer et al. report a 2.1 times lower level of co-infection with *B. burgdorferi* and *B. miyamotoi* (0.05% co-infection among 1,565 questing adult ticks) than expected by chance [48]. 

Tick surveillance demonstrates that *B. burgdorferi* s.l. is more prevalent than *B. miyamotoi,* approximating to a ratio of 10:1 or greater in several studies [5,6,48,49]. This is intriguing given that an American study suggests that both species infect ticks to equivalent levels achieving infection rates of around 2,000 spirochaetes for nymphs and 5,000 for adult ticks (sometimes higher in the case of *B. miyamotoi*) [6]. A European study, which quantified by PCR* B. miyamotoi* in feeding ticks removed from humans, even reported considerably higher borrelial tick loads by *B. miyamotoi* (mean count of 2.1 x 10^5^) than by *B. burgdorferi* s.l. (with a mean of 4.5 × 10^3^ for *B. afzelii* and 2.7 × 10^3^ for *B. garinii*) [49]. Thus, infection prevalence among ticks is higher for *B. burgdorferi* s.l. [5,6,48,49], however, *B. miyamotoi*-infected ticks appear to have equivalent [6], or higher quantities of borreliae within their tissues [49].

The answer to why we observe higher prevalence for *B. burgdorferi* s.l. compared with *B. miyamotoi* in ticks, might reside in the superior ability for *B. burgdorferi* to persist in the skin of its vertebrate host, potentially extending the window of transmission to other feeding ticks. The failure to detect *B. miyamotoi* in the skin of erythema migrans patients corroborates this theory [15]. Moreover, studies of *Peromyscus leucopus* mice during *I. scapularis* nymphal questing times have shown that Lyme-associated borreliae resided in the skin of their host, while *B. miyamotoi* gave a higher blood burden with density counts five times higher than *B. burgdorferi* s.l [6]. While levels up to 251 spirochaetes of *B. miyamotoi* per mL blood have been observed, persistence in blood within an infected vertebrate appears comparable to *B. burgdorferi* s.l. [6,50].

The sympatric overlap of *B. burgdorferi* s.l. and *B. miyamotoi* begs the question as to how these closely related spirochaetes might interact. Remarkably, the prevalence of *B. miyamotoi* infection in ticks appears indifferent to variations in the habitat type, or to ecological influences that impact upon the prevalence of *B. burgdorferi* s.l. [19]. As nutritional requirements are likely to overlap, is there antagonism between these species, indifference or a more synergistic relationship? Competitive interactions have not been explored per se, however, emerging data suggest that these species appear indifferent to each other. A study of shared small rodent hosts suggests different seasonal peaks of infection, with *B. burgdorferi* s.l. rodent infection peaking in spring and being driven by nymphs, whereas *B. miyamotoi* rodent infection being predominant in summertime through larval ticks [51,52]. This separation might provide a means for both spirochaetes to co-exist [6]. Indeed, transovarial tick transmission of *B. miyamotoi* enables larval ticks to be infectious for their subsequent hosts. In contrast, tick larvae harbouring *B. burgdorferi* s.l. are rarely reported, suggesting infrequent vertical transmission in *B. burgdorferi *s.l. Among field-collected larvae, a study found *B. burgdorferi* s.l. in 0.62% as opposed to 2% for *B. miyamotoi* [53]. Some recent publications query whether previously reported low levels of *B. burgdorferi* s.l. vertical transmission constituted in fact detection of *B. miyamotoi* [54,55].

### Transmission to humans, magnitude of human infection and pathogenesis

Emerging evidence supports presence of *B. miyamotoi* in tick salivary glands [34], with a study finding that over 88% of second generation progeny of infected *I. scapularis* nymphal ticks had *B. miyamotoi* in their salivary glands, as seen with other relapsing fever spirochaetes in their soft tick vectors [56]. Experimental mouse (CD-1 outbred *Mus musculus* mice) transmission studies using ticks derived from a field collected population infected with the American strain of *B. miyamotoi*, have demonstrated 10% infection after 24hrs, rising to 31% at 48hrs and 63% by 72hrs [56]. Though not as rapid as the transmission of classical relapsing fever spirochaetes by their soft tick vectors [57], this demonstrates transmission of *B. miyamotoi* in the first day of tick attachment, contrasting with the days of attachment needed by Lyme borreliae to migrate from the tick midgut to salivary glands for transmission [57]. In this respect, in humans, transmission efficiency has been estimated at 8.3% [58], based upon a cohort of 24 persons bitten by PCR-positive ticks, of whom only two later developed compatible clinical disease [58]. By comparison, three of 68 (4.4%), humans bitten by *B. burgdorferi* s.l. infected ticks, developed disease (erythema migrans) [58,59].

In terms of the magnitude of human infections with *B. miyamotoi*, this depends to some extent on the frequency of tick bites among people and on the prevalence of tick infection. Ixodid ticks are holarctic in distribution and assessments of tick bites vary by country and location. In the Netherlands, it was estimated that tick bites amount to 71,980 per million inhabitants (2007) [60]. In the Irkutsk region in Russia 2014, tick bites were estimated at 12,500 per million people [21]. The prevalence of infection among ticks ranges from 0.5 to 6% in many regions of the northern hemisphere. Taken together with a transmission rate of 8.3% [58], it is likely that human infections are underestimated [61].

In Yekaterinburg Province, Russia in 2009, it was estimated *B. miyamotoi* infections occur in 1 per 100,000 inhabitants [2]. Studies from the Netherlands suggest that some 36,000 humans are bitten each year by *B. miyamotoi* (European strain) infected ticks [9]. Serological studies from the US appear to corroborate human exposure [62,63]. Despite this, reports of human infections are comparatively few; for example, retrospective serological evaluation of sera from cases suspected for Lyme disease only detected 19 indigenous cases in Japan between 2013 and 2017 [64].

Borreliae as a group, are renowned for their ability to persist within humans. Relapsing fever *Borrelia* undergo an elaborate series of antigenic variation coupled with other mechanisms such as binding factor H and evading the components of the complement cascade, enabling their persistence in blood or their human host over time. Likewise, *B. miyamotoi* possesses variable membrane proteins (vmp) that could facilitate relapse with different antigenic variants [65], and has the ability to bind factor H and related proteins, thus evading host complement-mediated degradation [66-68]. Clinical reports of relapse with *B. miyamotoi* infection are described in the literature, however surprisingly, this appears less frequently than seen with classical relapsing fever infections [2,69]. 

The borreliae have proven neurotropism potential, using the nervous system as an immunologically protected niche enabling persistence in their vertebrate host [70,71]. The precise mechanisms by which spirochaetes exploit this niche are poorly understood, but animal studies have shown that relapsing fever *Borrelia* can survive for 270 days within this site, thus enabling greater *in vivo* persistence [70]. Neurological sequelae have been a feature of infection among immunocompromised individuals (see clinical presentations below) [72-74]. An *in vivo* infection model is urgently needed to unravel the pathogenesis of human *B. miyamotoi* infection. A severe combined immune deficiency (SCID) mouse model has been established [34], but has limitations to extrapolate to infection in immunocompetent humans.

### Clinical presentation

Clinical cases have been reported from Europe, Japan [4,64,75], China [13], Russia [2] and the US [76-78]. Infection with *B. miyamotoi* does not present with obvious hallmark signs. Instead, patients present with fever accompanied by non-specific influenza-like symptoms, such as chills, fatigue, headache, myalgia and arthralgia (Table 1) [9]. 

Although *B. miyamotoi* clusters within the relapsing fever group of spirochaetes, cases with the characteristic recurring febrile episodes interspersed with non-febrile intervals that typify classical relapsing fever have only been described sporadically [2,79]. In these, up to three febrile episodes have been recorded [2], however this might be an underestimation given that patients are typically managed with antimicrobial therapy upon diagnosis. A case who was retrospectively diagnosed following spontaneous recovery had a documented relapsing illness with two episodes, albeit with a lengthy 3-week afebrile period [69]. 

Furthermore, unlike relapsing fever spirochaetes, epistaxis, abortion, jaundice and major organ failure have not appeared as features of *B. miyamotoi* infection. Nevertheless, both *B. miyamotoi* and classical relapsing fever share fever, headaches, chills, myalgia, arthralgia, and nausea/vomiting. 

Some differences in clinical presentation have been noted between US and Russian cases, particularly regarding the presence of thrombocytopenia documented in approximately half of American cases [78], but not reported from those in Russia. This differential clinical presentation may be an artefact given the recent description of thrombocytopenia in a Japanese clinical case infected with an Asian *B. miyamotoi* strain [80], akin to those reported from Russian cases. 

In two studies, cases have been reported with erythema migrans [75,78], however it is likely that these had concomitant infection with *B. burgdorferi* s.l., thus representing co-infections. Indeed, one of these studies retrospectively sought presence of *B. miyamotoi* in sera from cases diagnosed with Lyme disease, whereas the other reported one case of erythema migrans among 51 *B. miyamotoi* patients who had an overall 14% co-infection rate with *B. burgdorferi* s.l. A larger study of 71 PCR-confirmed *B. miyamotoi* infected cases from Yekaterinburg, Russia, found only five of these cases with erythema migrans [22]. Analysis of blood for confirmation of pathogens disclosed that one case was co-infected with *B. burgdorferi* s.l., but it was concluded that the remaining cases with erythema migrans were realistically also co-infections given the poor sensitivity of PCR to detect Lyme disease using blood samples (as *B. burgdorferi* s.l. tends to be found in the skin) [22,49]. 

*Borrelia miyamotoi*, like other members of the borreliae have demonstrated their ability to result in neurological sequelae with descriptions of meningoencephalitis, albeit among immunocompromised individuals [64,72-74]. Unlike the acute febrile presentation described above, infections in these immunocompromised cases described to date, have shown a more insidious onset, often over several months. One case presented with memory deficits and disturbed gait, with lumbar puncture revealing pleocytosis and raised cerebrospinal fluid (CSF) protein [73]. 

### Diagnostics

For immunocompromised patients, diagnosis with microscopy has been used to a certain extent [72-74], sometimes combined with immunofluorescence [74]. For example, in three case reports on immunocompromised patients infected with *B. miyamotoi* [72-74], spirochaetes in the CSF were retrospectively detected by microscopy in one case [73]. For the other two cases, *B. miyamotoi* was also identified by microscopy, albeit after concentration of the CSF sample and either Giemsa or acridine orange staining [72,74]. For all three cases, however, microscopy was complemented with PCR for diagnosis confirmation [72-74].

Generally, for overall patients, diagnostic approaches depend on the stage and duration of infection. During the acute phase of infection, the presence of the spirochaete in blood and CSF can been demonstrated using PCR and microscopy. The success of such methods rapidly reduces from the fourth day of disease correlated with depletion in the spirochaetal blood counts [22]. Clinical reports of relapse are described in the literature [2,69]. If this occurs, it is likely that direct detection using PCR or microscopy might again be valuable. Use of concentration methods can improve the diagnostic sensitivity of these techniques [72,74,81]. Later in the clinical course, serology is the mainstay diagnostic option. 

In terms of PCR/molecular diagnostics during acute stages of infection − and for assessment of non-human vertebrates or ticks −, real-time PCR assays for *B. miyamotoi* based upon either 16S rDNA [6] or the flagellin gene target have been described. The effectiveness of such assays for detection of the newly described *B. miyamotoi* variants nevertheless remains to be established [82]. Given the sympatric nature of *B. miyamotoi* and its closely related Lyme-associated borreliae, a logical strategy would be to utilise a multiplex assay able to screen for both pathogens simultaneously. A multiplex approach is also probably better suited to a diagnostic setting to avoid multistep methods and reduce contamination risks that arise at each step. Some studies have suggested assays that differentiate conditions with overlapping clinical presenting features, such as *B. miyamotoi* infection and anaplasmosis [77] and have thus produced multiplex PCR assays to differentiate these infections [83].

Concerning serology, many studies have used the glycerophosphoryl diester phosphodiesterase (GlpQ) antigen expressed by members of the relapsing fever borreliae, but absent from *B. burgdorferi* s.l. [84]. Assays based upon GlpQ will not be *B. miyamotoi*-specific, but in areas where other members of the relapsing fever borreliae are not prevalent, such assays can be useful for population surveys and diagnosis on non-acute infections [85]. Besides the difficulties of GlpQ assays to distinguish *B. miyamotoi* from other relapsing fever borreliae, it is noteworthy that homologous proteins have been reported from both *Klebsiella pneumoniae* and *Salmonella enterica* [86]. In addition to lack of specificity, other reported limitations with the GlpQ antigen-based approach have been poor diagnostic sensitivity, with ability to only detect 28 of 36 convalescent samples from established cases [78]. 

As the use of vmps as antigens has also been explored for serodiagnosis [87], combinations of GlpQ together with mixtures of highly immunogenic vmps derived from *B. miyamotoi* have been evaluated as a way to improve the diagnostic efficacy [88]. This involved a comprehensive series of 182 PCR-confirmed Russian patients who were followed with sequential sera collected over several months post-infection. Notably, combinations of antigens provided superior sensitivity and/or specificity, with diagnostic titres for IgM, which were reached from 11 to 20 days post-disease onset and for IgG, from 21 to 50 days. This study used blood donor controls and additionally, controls with tick-borne encephalitis recruited from the same geographical region together with controls without tick exposure. Use of the combined antigens resulted in a sensitivity of 94.7% and specificity of 96.6% for IgM from 11 to 20 days post clinical presentation, thus providing important improvements over previous assays [88]. Assessment of duration of serological reactivity in seven of the 182 patients, showed that the IgM response waned within a year, while half (4/7) remained seropositive for IgG a year following disease. All individuals had been prescribed antimicrobial therapy.

On a cautionary note, the C6 ELISA used for diagnosing Lyme disease may additionally be positive in those infected by *B. miyamotoi* [69]. Furthermore, when using serological tests to detect *B. miyamotoi* in areas where multiple spirochaetes are endemic, the possibility of serological cross-reactivity between other relapsing fever borreliae such as *B. hermsii* should be kept in mind as this may obscure diagnosis [64,85].

Given the increasing recognition of co-infections among those with tick exposure, it might be prudent to take a more holistic approach and screen for a broader range of tick-borne pathogens than just borreliae [89]. Some diagnostic centres have taken this approach, but many do not yet have the resources for more comprehensive tick-borne pathogen screening.

Isolation of borreliae is always a challenge as these microbes are particularly fastidious to cultivate, requiring complex liquid medium. Isolation is typically confirmed by dark field microscopy. The Barbour–Stoenner–Kelly (BSK) commercially-available medium (BSK-H) used for *B. burgdorferi* s.l. is unreliable for growth of relapsing fever spirochaetes (data not shown) and when used for *B. miyamotoi*, is unable to sustain passage of this organism [90]. Growth of *B. miyamotoi* has been achieved using modifications of Kelly–Pettenkofer medium (MKP) [16,91] or in a variation of BSK described as BSK-M [4]. Other studies have used media modifications with inclusion of 50% serum in order to cultivate these spirochaetes [92].

Propagation of isolates has additionally been achieved using inoculation of SCID mice. Typically, these will show spirochaetes in blood films between 7 and 14 days post-infection [34].

### Treatment

Therapy for *B. miyamotoi* infection has typically followed guidelines used for treatment of Lyme borreliosis. Only a few cultivable strains have been recovered to date, restricting evaluation of different clinical management regimes. Moreover limited *in vitro* susceptibility testing has been undertaken to verify the efficacy of different therapeutic protocols [93]. Koetsveld and co-authors, noted resistance to amoxicillin *in vitro* (16–128 mg/L) using two isolates of *B. miyamotoi* [93]. Interestingly, this feature was also shared by the relapsing fever spirochaete *B. hermsii* that was assessed in parallel. Despite these *in vitro* findings, a patient treated with amoxicillin (and sultamicillin) responded without complications [80]. No treatment failures have been reported to date, thus it is probable that the hypothesised susceptibility profile being analogous to the Lyme-associated species is supported. Standard methods are not applicable for evaluation of the susceptibility testing of borreliae given their need for liquid cultivation, microaerophilic conditions and coupled with their slow mean generation time [94-96]. Akin to other members of the genus and spirochaetal infections in general, a proportion of patients may develop a Jarisch–Herxheimer reaction (JHR) associated with a sudden exacerbation of clinical signs upon onset of treatment [97]. Though reported, JHR does not appear frequently for cases of acute *B. miyamotoi* infection [2,74].

### Future research directions

Much of the data so far arise from studies designed and funded to look either at tick-borne diseases or more specifically Lyme-associated borreliae. Though valuable, these studies might be biased and not reflect some of the different ecological driving factors underpinning the observed epidemiology of this spirochaete. As such, we still need to more specifically address *B. miyamotoi* epidemiology further. This is important both for risk assessment and for application of control/intervention strategies. Indeed, many questions remain regarding our understanding of the pathobiology of this spirochaete (Table 3).

**Table 3:** Unanswered questions regarding the pathobiology of *Borrelia miyamotoi*, 2018

What is the global epidemiological picture of *B. miyamotoi* infection?
Are the different spirochaetal variants restricted among certain tick species?
What is the ecology of this spirochaete?
What is the contribution of high incidence vertebrate species such as wild turkeys towards maintaining the ecological niche for this spirochaete?
What are the consequences of other pathogens present within ticks (including other *Borrelia*) upon the survival, persistence and transmissibility of *B. miyamotoi*?
Do different strains show differential virulence within susceptible species?
What are the full range of clinical consequences within humans?
What are human risk factors for development of clinical disease above and beyond being immunocompromised?
Does blood transfusion present a substantive risk for infection?
What is the best diagnostic approach to take, using which sample types and at what time point during infection?
What is the best regime for therapeutic management of cases?

Future epidemiological studies need to consider the different genotypes of* B. miyamotoi*, particularly in areas of geographical overlap such as in Estonia and Russia [2,11]. Ability to assess the epidemiology of *B. miyamotoi* has been enhanced by the development of several multiplex PCR diagnostic methods providing a more cost-effective means for high throughput screening of samples [6,82,83]. These approaches, which are tailored to detect genotypes already described, however run the risk of missing hitherto undescribed variants. 

Indeed, while three genotypes of *B. miyamotoi* (American, Asian and European) have been previously described, each associated with different tick vectors (Table 2) [3], recent studies have found more diversity within the *B. miyamotoi* species [10,12,28]. Future studies may shed light on how diverse *B. miyamotoi* genotype strains and variants behave. For example, it has not been comprehensively addressed if each of the *B. miyamotoi* genotypes or variants and their respective tick hosts are equally competent for transovarial transmission. Our knowledge to date is based upon studies that typically have only assessed one strain and tick species [33,54]. This information is currently extrapolated to others, but not underpinned by rigorous scientific enquiry.

Despite larger diversity recognised among *B. miyamotoi* strains, the species appears to be less heterogeneous than the *B. burgdorferi* s.l. complex, for which representatives sympatrically overlap with *B. miyamotoi*. Although it needs to be evaluated how strict new *B. miyamotoi* variants are to certain tick species, the variants identified to date have been found to be generally restricted to separate tick species, supporting the idea that, like for other *B. miyamotoi* spirochaetes, ticks still serve as both reservoirs and vectors for these [19]. Conversely, for the Lyme-associated species, ticks act mainly as vectors, while a plethora of vertebrates serve as reservoirs for horizontal infection, potentially driving diversity within this complex.

This being said, vertebrates also play a role in the ecology of *B. miyamotoi*. In this respect, it is notable that high infection rates were reported in turkeys [41]. Studies of avian vertebrates and their role in the ecology of *B. miyamotoi* have largely excluded ground foraging gallinaceous species, which is surprising given their established role as a reservoir for *B. burgdorferi* s.l [98]. Looking into these avian species may provide missing pieces of the jigsaw of understanding the ecological niche of this spirochaete.

Coinfections by *B. burgdorferi* and *B. miyamotoi* have been observed in both vertebrates and ticks. Within ticks, a further level of complexity arises from the growing appreciation of how different microbes might facilitate survival or transmission. An ecological synergy is proposed whereby *B. burgdorferi* s.l. and *Babesia microti* benefit each other [99], but currently we have no knowledge of such interactions for *B. miyamotoi*. Importantly, we should consider how other pathogens present within ticks (including other *Borrelia*) will influence the survival, persistence and transmissibility of *B. miyamotoi*.

To anticipate human infection, consideration of the tick species likely to be encountered is important. An example might be the detected presence of *B. miyamotoi* within *I. dentatus* ticks that preferentially feed upon birds or lagomorphs and thus present a reduced risk of human infection [31]. Such information would enable instigation of risk prediction, modelling and targeted intervention approaches where justified. 

Blood transfusion has been hypothesised as a risk factor for *B. miyamotoi* infection. Experimental studies have demonstrated the ability of *B. miyamotoi* to survive under conditions used for storage of blood transfusion products [100], raising concern that this could provide an additional source of infection (Table 3). Though theoretically possible, clinical cases presenting with acute signs, typically fever and associated non-specific influenza-like features, unlikely would present to donate blood [101]. Quantification of the numbers of spirochaetes present during human infection suggests that levels peak at ca 10^3^−10^4^ *B. miyamotoi* copies per mL during the second to third day of illness, then rapidly wane until day eight [22]. These authors suggested that PCR diagnosis was unreliable after the fourth day of clinical signs which would suggest low risk of transfusion related infection.

An enigma of our current understanding is why we fail to see a correlation between clinical cases of *B. miyamotoi* infection and tick infection prevalence. Human cases have occurred in some areas with relatively low tick infection prevalence, like Yekaterinburg, Russia, while in other countries or areas with higher prevalence in ticks, such as Mongolia or Hannover in Germany, no cases were reported. The reasons are likely to be multifactorial. Tick factors such as feeding preferences or the role of vertebrate hosts in intensifying or negatively impacting transmission could perhaps account for the observations. Another explanation might be differential virulence in humans of the strains involved, as it remains to be solved if different strains/genotypes show differential virulence. Last, the absence of an evident relation between tick and human infection rates could also possibly result from lack of diagnostic ability, proximity to those with active research interest in tick-borne disease, or cases missed due to unspecific symptoms. The clinical features of *B. miyamotoi* indeed lack a diagnostic hallmark, and can potentially be misdiagnosed as granulocytic anaplasmosis [77,102]. Given these difficulties to recognise *B. miyamotoi *infections, it is likely that cases are under-reported. 

While spontaneous resolution of an infection with *B. miyamotoi* can occur without antimicrobial intervention [69,103], some clinical studies have noted severe infection, with considerable proportions of cases being hospitalised (24% [78]). Because when cases are diagnosed, they are likely to be promptly treated, the long-term consequences of human infection remain to be determined. While case studies of immunocompromised individuals report notable clinical features [64,72-74], the other risk factors that might influence the clinical progression towards disease are also not clear. Future detailed epidemiological studies of cases detected will enable a more complete clinical picture to unfold.

We also need to be aware of unusual presentations resulting from potential co-infection. The areas reporting cases are likely endemic for a variety of different tick-borne diseases. Clinically, presence of one tick-borne pathogen can obscure the concomitant presence of another. Consequently, it is a priority that those suspected of *B. miyamotoi* infection be also comprehensively screened for other tick-borne pathogens.

Diagnostic methods are nevertheless still widely considered ‘research tests’ and thus not generally available in more routine clinical settings. When these are possible, a delay in considering *B. miyamotoi* infection might further complicate their interpretation, due to the poorly delineated clinical course of human infection. A further problem may be the variability of strains encountered which may affect diagnostic results.

Another problem relates to what the most appropriate diagnostic sample is. Ticks removed from patients have limited diagnostic value in that transmission may not have occurred and this might only be one of several ticks that might have bitten the individual in question. During acute infection, blood samples are a key sample to collect, however samples should be taken as early as possible and certainly before commencement of antimicrobial therapy. Collection of CSF is valuable in cases showing neurological features. This might require concentration of the specimen to improve diagnostic sensitivity [72,74]. Serum samples for serological investigation should be collected at all stages, with early sera used for assessment of seroconversion or increasing titre, and later samples for retrospective studies of prior exposure. 

As for the therapeutic management of cases, a more comprehensive *in vitro* evaluation will be possible to guide this, once more isolates become available representing the diversity within this species. 

## Conclusion

Our understanding of *B. miyamotoi* and its ecology and infection potential have only recently started to unfold. The incidence of *B. miyamotoi* infection in humans is poorly explored, hampered by the lack of awareness and appropriate diagnostics. Our understanding of the clinical features of infection currently suggest a relatively mild infectious course without long-lasting sequelae for the majority of infected individuals, but with the caveat of being able to cause severe disease in the immunocompromised [72,74,86]. Current incidence is likely to be grossly under-reported, suggested by predicted tick bite exposure and tick prevalence data, as well as through the absence of a clinical presentation hallmark, making assessment of the impact of infection challenging.
